# Supplementary material for: Survey, Detection, Characterization of Papaya Ringspot Virus from Southern India and Management of Papaya Ringspot Disease
Source: Pathogens. 2023 Jun 11;12(6):824. doi: 10.3390/pathogens12060824 (PMC10301999; doi:10.3390/pathogens12060824)
Supplement: Supplementary file 1 [file pathogens-12-00824-s001.zip › Supplementary Figures.pdf]

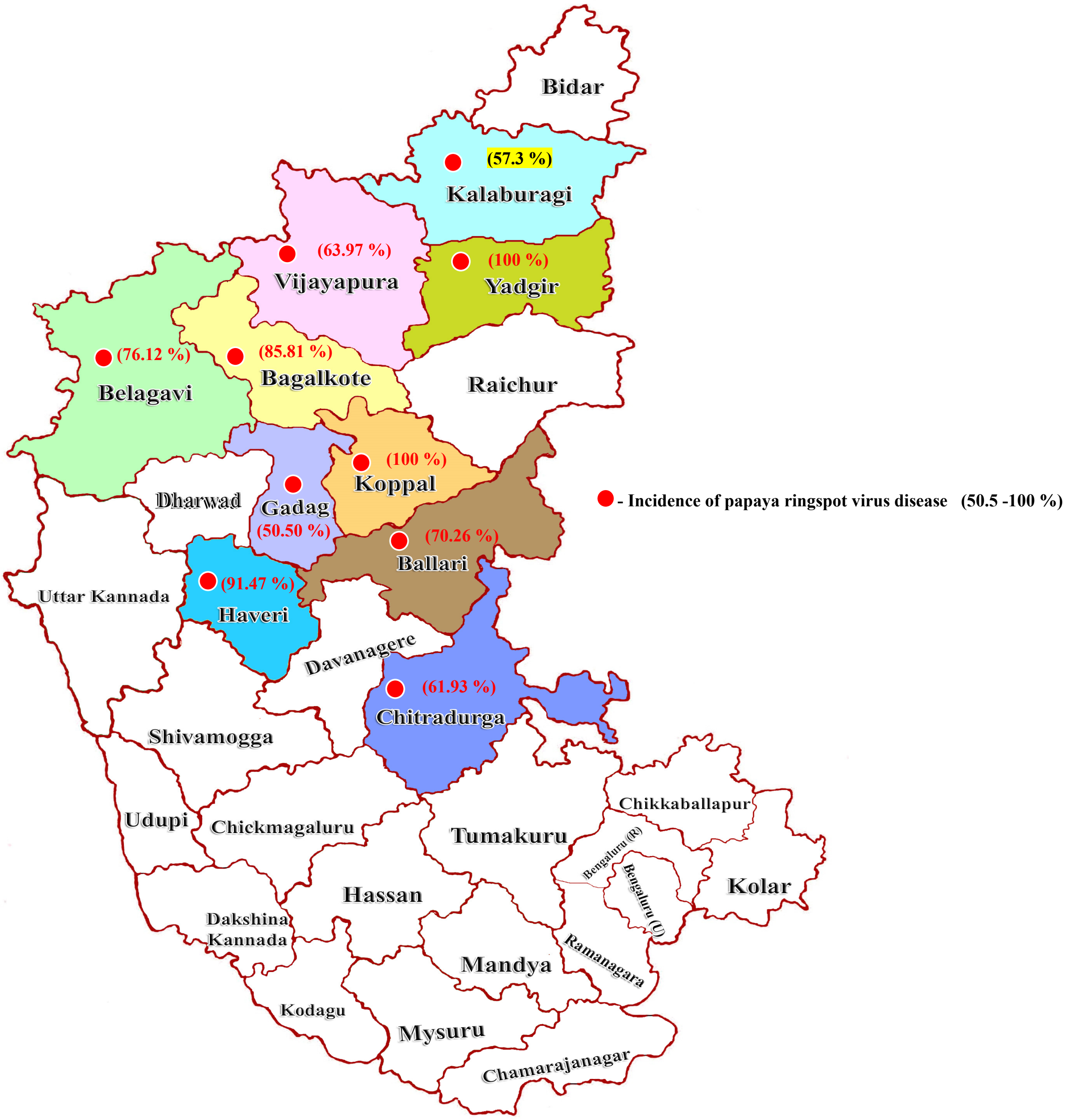

Figure S1. Status of papaya ringspot disease in major papaya growing districts of Karnataka state, India.

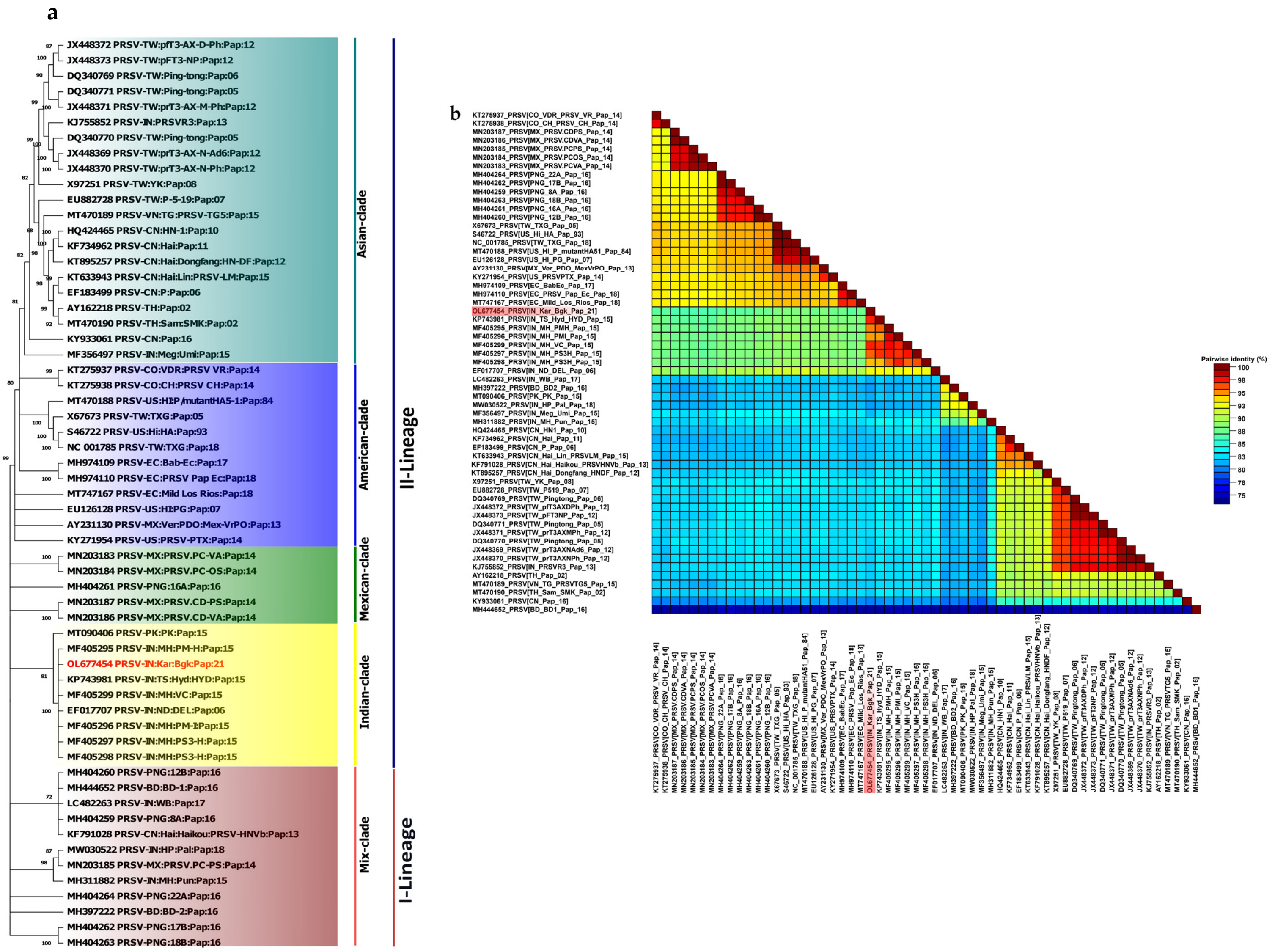

Figure S2: Phylogenic tree showing the relationship of PRSV-BGK (OL677454) with other selected PRSV isolates based on their complete genome of the nt sequence (the number of each node indicates 60 percentage bootstrap value with 1000 replicates) (a) and graphical representation of percent pair wise genome scores and nt identity matrix using SDT (b).
