# Supplementary material for: Survey, Detection, Characterization of Papaya Ringspot Virus from Southern India and Management of Papaya Ringspot Disease
Source: Pathogens. 2023 Jun 11;12(6):824. doi: 10.3390/pathogens12060824 (PMC10301999; doi:10.3390/pathogens12060824)
Supplement: Supplementary file 1 [file pathogens-12-00824-s001.zip › Supplementary Tables.pdf]

Table S1: Primers and PCR amplification conditions used for detection and characterization of PRSV infecting papaya and other viruses.

Primer	Oligonucleotides (5′-3′)	Length (nt)	PCR condition: Initial-denaturation	No. of cycles	Denaturation	Annealing	Extension	Final extension	Reference
**Papaya ringspot virus primers**									
MB 11A	GGATCCATGTCCAAAAATGAAGCTGTGGATGCT	33	94°C for 5 min	35	94°C for 2 min	55°C for 1 min	72°C for 1 min	72°C for 10 min	Bateson, *et al*. (1994)
MB 11B	TCAATGGCGCATACCCAGGAGAGT	24							
PRSV878S –F	CACCTGGCACTCGAAGTTGGCAATGAAA	28	94°C for 5 min	35	94°C for 30 Sec	55°C for 30 Sec	72°C for 2 min	72°C for 7 min.	Ortiz-Rojas and Chaves-Bedoya (2017)
PRSV2746A-R	TTTCTTAGCTATGTACATTGAATCAGAC	28							
PRSV2289S –F	TCACTTCAACCCAGCTCTTACGTGTGAC	28							
PRSV3926A-R	TGCTTCAATTGTATATGCTCTGTGG	25							
PRSV4199S –F	TCGACGTGGACAAGAGTGACTGTGTTTA	28							
PRSV5924A–R	TCACTTTCAATCAGTTTATCAATCGTTA	28							
PRSV8961S –F	GTCATGGGGATATGGGGAGTTAACACA	27							
PRSVPOLYT-R	TTTTTTTTCTCTCATTCTAAGAGGCTCG	28							
PRSV3310S –F	GTTTTAGTAGAGCAATTATGTCGGAAAG	28	94°C for 5 min	35	94°C for 30 Sec	50.9°C for 30 Sec	72°C for 2 min	72°C for 7 min.	
PRSV4850A–R	TTTTCAATCGCGTTCCCCTCCATCAAA	27							
PRSV5355S –F	TGGAAGACATAAAGAGGGAATTGCACTA	28							
PRSV7195A–R	TTGCTCTGCTGTGGAATGGTGGAAAGTC	28							
PRSV1S -F	AAATAAAACATCTCAACACAACACAATTCAAAG	33	94°C for 5 min	35	94°C for 30 Sec	60°C for 30 Sec	72°C for 2 min	72°C for 7 min.	
PRSV1780A-R	TGCACACATGATCCGTTGGTTTTCT	25							
PRSV6777S –F	TGATTTAACACCACATAACCCACTCAAG	28							
PRSV8041A–R	TTTGTAAAACTTTGTCATGCCAACAGTC	28							
PRSV7497S –F	CAGGCAGATTGATGATTTAACTTGGAGC	28							
PRSV9389A–R	TCTCTATCTCTCTCTCCAGTTTTTGTGC	28							

**Milk vetch dwarf virus primers**									
M-F	CTAGTCAGCCATCCAATGGTG	21	94°C for 3 min	35	94°C for 30 Sec.	58°C for 30 Sec.	72°C for 1 min	72°C for 10 min.	Lal *et al.,* (2020)
M-R	GTGCAGGGTTTGATTGTCTGC	21							
**Papaya leaf distortion virus primers**									
PLDMV355-F	GGCATGTGGTTTATGATGCAAGGG	24	94°C for 3 min	35	94°C for 30 Sec.	55°C for 30 Sec.	72°C for 1 min.	72°C for 5 min.	Tuo *et al.,* (2014)
PLDMV355-R	GCTCCGTGTTCTCAGTCGCATT	22							
**Papaya mosaic virus primers**									
PapMV205-F	CCAAATTTGCCGCGTTCGACT	21	94°C for 3 min	35.	94°C for 30 Sec.	55°C for 30 Sec	72°C for 1 min.	72°C for 5 min	Tuo *et al.,* (2014)
PapMV205-R	GACCCAGAAATTTGGCCTTTGGTGATG	27							
**Zucchini yellow mosaic virus primers**									
ZYMV-F (CPF-Nocl)	CCATGGTCAGGCACTCARCCAACT	24	94°C for 5 min	35	94°C for 30 Sec	55°C for 30 Sec	72°C for 2 min	72°C for 7 min.	Villamor *et al.,* (2003)
ZYMV-R (CPR-Nocl)	CCATGGCTGCATTGRTTCACACCTAGYAAG	30							

Table S2. GenBank accession numbers of selected potyvirus sequences retrieved from database used in this study for analysis of PRSV isolate.

Sl. No.	Organism	Accession No.	Acronym
1.	*Papaya ringspot virus*-[Bangladesh:BD-1:Papaya:2016]	MH444652	PRSV-[BD:BD-1:Pap:16]
2.	*Papaya ringspot virus*-[Bangladesh:BD-2:Papaya:2016]	MH397222	PRSV-[BD:BD-2:Pap:16]
3.	*Papaya ringspot virus*-[China:Hainan:Dongfang:HN-DF:Papaya:2012]	KT895257	PRSV-[CN:Hai:Dongfang:HN-DF:Pap:12]
4.	*Papaya ringspot virus*-[China:Hainan:Haikou:PRSV-HNVb:Papaya:2013]	KF791028	PRSV-[CN:Hai:Haikou:PRSV-HNVb:Pap:13]
5.	*Papaya ringspot virus*-[China:Hainan:Lingshui:PRSV-LM:Papaya:2015]	KT633943	PRSV-[CN:Hai:Lin:PRSV-LM:Pap:15]
6.	*Papaya ringspot virus*-[China:Hainan:Papaya:2011]	KF734962	PRSV-[CN:Hai:Pap:11]
7.	*Papaya ringspot virus*-[China:HN-1:Papaya:2010]	HQ424465	PRSV-[CN:HN-1:Pap:10]
8.	*Papaya ringspot virus*-[China:P:Papaya:2006]	EF183499	PRSV-[CN:P:Pap:06]
9.	*Papaya ringspot virus*-[China:Papaya:2016]	KY933061	PRSV-[CN:Pap:16]
10.	*Papaya ringspot virus*-[Colombia:Campo Hermoso:PRSV_CH:Papaya:2014]	KT275938	PRSV-[CO:CH:PRSV_CH:Pap:14]
11.	*Papaya ringspot virus*-[Colombia:Villa del Rosario:PRSV_VR:Papaya:2014]	KT275937	PRSV-[CO:VDR:PRSV_VR:Pap:14]
12.	*Papaya ringspot virus*-[Ecuador:Bab-Ec:Papaya:2017]	MH974109	PRSV-[EC:Bab-Ec:Pap:17]
13.	*Papaya ringspot virus*-[Ecuador:Mild_Los_Rios:Papaya:2018]	MT747167	PRSV-[EC:Mild_Los_Rios:Pap:18]
14.	*Papaya ringspot virus*-[Ecuador:PRSV_Pap_Ec:Papaya:2018]	MH974110	PRSV-[EC:PRSV_Pap_Ec:Pap:18]
15.	*Papaya ringspot virus*-[India: Maharashtra:PS3-H:Papaya:2015]	MF405297	PRSV-[IN:MH:PS3-H:Pap:15]
16.	*Papaya ringspot virus*-[India: Maharashtra:VC:Papaya:2015]	MF405299	PRSV-[IN:VC:Pap:15]
17.	*Papaya ringspot virus*-[India:HimachalPradesh:Palampur:Papaya:2018]	MW030522	PRSV-[IN:HP:Pal:Pap:18]
18.	*Papaya ringspot virus*-[India:Maharashtra:PM-H:Papaya:2015]	MF405295	PRSV-[IN:MH:PM-H:Pap:15]
19.	*Papaya ringspot virus*-[India:Maharashtra:PM-I:Papaya:2015]	MF405296	PRSV-[IN:MH:PM-I:Pap:15]
20.	*Papaya ringspot virus*-[India:Maharashtra:PS3-H:Papaya:2015]	MF405298	PRSV-[IN:MH:PS3-H:Pap:15]
21.	*Papaya ringspot virus*-[India:Maharashtra:Pune:Papaya:2015]	MH311882	PRSV-[IN:MH:Pun:Pap:15]
22.	*Papaya ringspot virus*-[India:Meghalaya:Umiam:Papaya:2015]	MF356497	PRSV-[IN:Meg:Umi:Pap:15]
23.	*Papaya ringspot virus*-[India:New Delhi:DEL:Papaya:2006]	EF017707	PRSV-[IN:ND:DEL:Pap:06]
24.	*Papaya ringspot virus*-[India:Rajasthan:PRSVR3:Papaya:2013]	KJ755852	PRSV-[IN:PRSVR3:Pap:13]
25.	*Papaya ringspot virus*-[India:Telangana:Hyderabad:HYD:Papaya:2015]	KP743981	PRSV-[IN:TS:Hyd:HYD:Pap:15]
26.	*Papaya ringspot virus*-[India:West Bengal:WB:Papaya:2017]	LC482263	PRSV-[IN:WB:Pap:17]
27.	*Papaya ringspot virus*-[Mexico:PRSV.CD-PS:Papaya:2014]	MN203187	PRSV-[MX:PRSV.CD-PS:Pap:14]
28.	*Papaya ringspot virus*-[Mexico:PRSV.CD-VA:Papaya:2014]	MN203186	PRSV-[MX:PRSV.CD-VA:Pap:14]
29.	*Papaya ringspot virus*-[Mexico:PRSV.PC-OS:Papaya:2014]	MN203184	PRSV-[MX:PRSV.PC-OS:Pap:14]
30.	*Papaya ringspot virus*-[Mexico:PRSV.PC-PS:Papaya:2014]	MN203185	PRSV-[MX:PRSV.PC-PS:Pap:14]

31.	*Papaya ringspot virus*-[Mexico:PRSV.PC-VA:Papaya:2014]	MN203183	PRSV-[MX:PRSV.PC-VA:Pap:14]
32.	*Papaya ringspot virus*-[Mexico:Veracruz:Paso de Ovejas:Mex-VrPO:2003]	AY231130	PRSV-[TH:Ver:PDO:Mex-VrPO:Pap:13]
33.	*Papaya ringspot virus*-[Pakistan:PK:Papaya:2015]	MT090406	PRSV-[PK:PK:Pap:15]
34.	*Papaya ringspot virus*-[Papua New Guinea:12B:Papaya:2016]	MH404260	PRSV-[PNG:12B:Pap:16]
35.	*Papaya ringspot virus*-[Papua New Guinea:16A:Papaya:2016]	MH404261	PRSV-[PNG:16A:Pap:16]
36.	*Papaya ringspot virus*-[Papua New Guinea:17B:Papaya:2016]	MH404262	PRSV-[PNG:17B:Pap:16]
37.	*Papaya ringspot virus*-[Papua New Guinea:18B:Papaya:2016]	MH404263	PRSV-[PNG:18B:Pap:16]
38.	*Papaya ringspot virus*-[Papua New Guinea:22A:Papaya:2016]	MH404264	PRSV-[PNG:22A:Pap:16]
39.	*Papaya ringspot virus*-[Papua New Guinea:8A:Papaya:2016]	MH404259	PRSV-[PNG:8A:Pap:16]
40.	*Papaya ringspot virus*-[Taiwan Ping-tong:Papaya:2005]	DQ340771	PRSV-[TW:Ping-tong:Pap:05]
41.	*Papaya ringspot virus*-[Taiwan:P-5-19:Papaya:2007]	EU882728	PRSV-[TW:P-5-19:Pap:07]
42.	*Papaya ringspot virus*-[Taiwan:pfT$_3$-AX-D-Ph:Papaya:2012]	JX448372	PRSV-[TW:pfT$_3$-AX-D-Ph:Pap:12]
43.	*Papaya ringspot virus*-[Taiwan:pFT$_3$-NP:Papaya:2012]	JX448373	PRSV-[TW:pFT$_3$-NP:Pap:12]
44.	*Papaya ringspot virus*-[Taiwan:Ping-tong:Papaya:2005]	DQ340770	PRSV-[TW:Ping-tong:Pap:05]
45.	*Papaya ringspot virus*-[Taiwan:Ping-tong:Papaya:2006]	DQ340769	PRSV-[TW:Ping-tong:Pap:06]
46.	*Papaya ringspot virus*-[Taiwan:prT$_3$-AX-M-Ph:Papaya:2012]	JX448371	PRSV-[TW:prT$_3$-AX-M-Ph:Pap:12]
47.	*Papaya ringspot virus*-[Taiwan:prT$_3$-AX-N-Ad6:Papaya:2012]	JX448369	PRSV-[TW:prT$_3$-AX-N-Ad6:Pap:12]
48.	*Papaya ringspot virus*-[Taiwan:prT$_3$-AX-N-Ph:Papaya:2012]	JX448370	PRSV-[TW:prT$_3$-AX-N-Ph:Pap:12]
49.	*Papaya ringspot virus*-[Taiwan:Taichung:Papaya:2005]	X67673	PRSV-[TW:TXG:Pap:05]
50.	*Papaya ringspot virus*-[Taiwan:Taichung:Papaya:2018]	NC_001785	PRSV-[TW:TXG:Pap:18]
51.	*Papaya ringspot virus*-[Taiwan:YK:Papaya:2008]	X97251	PRSV-[TW:YK:Pap:08]
52.	*Papaya ringspot virus*-[Thailand:Papaya:2002]	AY162218	PRSV-[TH:Pap:02]
53.	*Papaya ringspot virus*-[Thailand:Samutsakhon:SMK:Papaya:2013]	MT470190	PRSV-[TH:Sam:SMK:Pap:02]
54.	*Papaya ringspot virus*-[USA:Hawaii:HA:Papaya:1993]	S46722	PRSV-[US:Hi:HA:Pap:93]
55.	*Papaya ringspot virus*-[USA:Hawaii:P/mutant HA 5-1:Papaya:1984]	MT470188	PRSV-[US:HI:P/mutantHA5-1:Pap:84]
56.	*Papaya ringspot virus*-[USA:Hawaii:PG:Papaya:2007]	EU126128	PRSV-[US:HI:PG:Pap:07]
57.	*Papaya ringspot virus*-[USA:PRSV-PTX:Papaya:2014]	KY271954	PRSV-[US:PRSV-PTX:Pap:14]
58.	*Papaya ringspot virus*-[Viet Nam:Tien Giang:PRSV- TG5:Papaya:2015]	MT470189	PRSV-[VN:TG:PRSV-TG5:Pap:15]

Table S3. Details of the treatment combinations of insecticides and biorationals for the management of PRSD under field conditions

Treatment	1st Spray at 30 DAT*	2nd Spray at 60 DAT	3rd Spray at 90 DAT	4th Spray at 120 DAT	5th Spray at 150 DAT	6th Spray at 180 DAT	7th Spray at 210 DAT	8th Spray at 240 DAT
T_1	Tolfenpyrad 15% EC 1 ml/l followed by micronutrients**	Imidacloprid 17.8% SL 0.2 ml/l followed by micronutrients	Thiacloprid 21.7 SC 1 ml/l followed by micronutrients	Dinotefuran 20 % SG 0.5g/l followed by micronutrients	Tolfenpyrad 15% EC 1 ml/l followed by micronutrients	Imidacloprid 17.8% SL 0.2 ml/l followed by micronutrients	Thiacloprid 21.7 SC 1 ml/l followed by micronutrients	Dinotefuran 20 % SG 0.5g/l followed by micronutrients
T_2	1% Neem oil 10 ml/l followed by micronutrients	Pongamia oil 10 ml/l followed by micronutrients	Groundnut oil 10 ml/l followed by micronutrients	Mineral oil 10 ml/l followed by micronutrients	1% Neem oil 10 ml/l followed by micronutrients	Pongamia oil 10 ml/l followed by micronutrients	Groundnut oil 10 ml/l followed by micronutrients	Mineral oil 10 ml/l followed by micronutrients
T_3	Seaweed extract 4ml/l followed by micronutrient	Seaweed extract 4ml/l followed by micronutrient	Seaweed extract 4ml/l followed by micronutrient	Seaweed extract 4ml/l followed by micronutrient	Seaweed extract 4ml/l followed by micronutrient	Seaweed extract 4ml/l followed by micronutrient	Seaweed extract 4ml/l followed by micronutrient	Seaweed extract 4ml/l followed by micronutrient
T_4	Tolfenpyrad 15% EC 1 ml/l followed by micronutrients	1% Neem oil 10 ml/l followed by micronutrients	Imidacloprid 17.8% SL 0.2 ml/l followed by micronutrients	Pongamia oil 10 ml/l followed by micronutrients	Thiacloprid 21.7 SC 1 ml/l followed by micronutrients	Groundnut oil 10 ml/l followed by micronutrients	Dinotefuran 20 % SG 0.5g/l followed by micronutrients	Mineral oil 10 ml/l followed by micronutrients
T_5	Tolfenpyrad 15% EC 1 ml/l followed by micronutrients	Seaweed extract 4ml/l followed by micronutrients	Imidacloprid 17.8% SL 0.2 ml/l followed by micronutrients	Seaweed extrac- 4ml/l followed by micronutrients	Thiacloprid 21.7 SC 1 ml/l followed by micronutrients	Seaweed extract 4ml/l followed by micronutrients	Dinotefuran 20 % SG 0.5g/l followed by micronutrients	Seaweed extract 4ml/l followed by micronutrients
T_6	1% Neem oil 10 ml/l followed by micronutrients	Seaweed extract 4ml/l followed by micronutrients	Pongamia oil 10 ml/l followed by micronutrients	Seaweed extract 1gm/l followed by micronutrients	Groundnut oil 10 ml/l followed by micronutrients	Seaweed extract 4ml/l followed by micronutrients	Mineral oil 10 ml/l followed by micronutrients	Seaweed extract 4ml/l followed by micronutrients
T_7	Tolfenpyrad 15% EC 1 ml/l followed by micronutrients	1%Neem oil 10 ml/l followed by micronutrients	Seaweed extract 4ml/l followed by micronutrients	Seaweed extract 4ml/l followed by micronutrients	Imidacloprid 17.8% SL 0.2 ml/l followed by micronutrients	Groundnut oil 10 ml/l followed by micronutrients	Seaweed extract 4ml/l followed by micronutrients	Seaweed extract 4ml/l followed by micronutrients
T_8	Micronutrients*	Micronutrients	Micronutrients	Micronutrients	Micronutrients	Micronutrients	Micronutrients	Micronutrients
T_9	Control (No spray)							

*DAT: Day After Transplanting, ** Zn-3.0%, Fe-2.0%, Mn-1.0%, B-0.50%, Mg-2.0%

Table S4. Details of the IDM modules for integrated management of PRSD under field conditions

Treatment	1st Spray at 20 DAT*	2nd Spray at 40 DAT	3rd Spray at 60 DAT	4th Spray at 80 DAT	5th Spray at 100 DAT	6th Spray at 120 DAT
M_1	Tolfenpyrad 15EC 1 ml/l followed by micronutrients **	Imidacloprid 17.8% SL 0.2 ml/l followed by micronutrients	Thiacloprid 21.7 SC 1 ml/l followed by micronutrients	Dinotefuran 20 % SG 0.5g/l followed by micronutrients	Tolfenpyrad 15EC 1 ml/l followed by micronutrients	Imidacloprid 17.8% SL 0.2 ml/l followed by micronutrients
M_2	Tolfenpyrad 15EC 1 ml/l followed by micronutrients	Seaweed extract 4ml/l followed by micronutrients	Imidacloprid 17.8% SL 0.2 ml/l followed by micronutrients	Seaweed extract 4ml/l followed by micronutrients	Thiacloprid 21.7 SC 1 ml/l followed by micronutrients	Seaweed extract 4ml/l followed by micronutrients
M_3	Tolfenpyrad 15EC 1 ml/l followed by micronutrients	Seaweed extract 4ml/l followed by micronutrients	Imidacloprid 17.8% SL 0.2 ml/l followed by micronutrients	Seaweed extract 4ml/l followed by micronutrients	Thiacloprid 21.7 SC 1 ml/l followed by micronutrients	Seaweed extract 4ml/l followed by micronutrients
M_4 (UHS POP)	Dimethoate 30 % EC 1.7ml/l followed by 1% neem oil 10 ml/l	Neam Leaf extract 1500 ppm 2 ml/l	Oxydemeton methyl 25% EC 1.5ml/l followed by 1% groundnut oil 10 ml/l	Neam leaf extract 1500 ppm 2 ml/l	Imidacloprid 17.8% SL 0.25ml/l followed by 1% neem oil 10 ml/l	Neam leaf extract 1500 ppm 2 ml/l

Treatment	7th Spray at 140 DAT	8th Spray at 160 DAT	9th Spray at 180 DAT	10th Spray at 200 DAT	11th Spray at 220 DAT	12th Spray at 240 DAT
M_1	Thiacloprid 21.7 SC 1 ml/l followed by micronutrients	Dinotefuran 20 % SG 0.5g/l followed by micronutrients	Tolfenpyrad 15EC 1 ml/l followed by micronutrients	Imidacloprid 17.8% SL 0.2 ml/l followed by micronutrients	Thiacloprid 21.7 SC 1 ml/l followed by micronutrients	Dinotefuran 20 % SG 0.5g/l followed by micronutrients
M_2	Dinotefuran 20 % SG 0.5g/l followed by micronutrients	Seaweed extract 4ml/l followed by micronutrients	Tolfenpyrad 15EC 1 ml/l followed by micronutrients	Seaweed extract 4ml/l followed by micronutrients	Imidacloprid 17.8% SL 0.2 ml/l followed by micronutrients	Seaweed extract 4ml/l followed by micronutrients
M_3	Dinotefuran 20 % SG 0.5g/l followed by micronutrients	Seaweed extract 4ml/l followed by micronutrients	1%Neem oil 10 ml/l followed by micronutrients	Seaweed extract 4ml/l followed by micronutrients	Groundnut oil 10 ml/l followed by micronutrients	Seaweed extract 4ml/l followed by micronutrients
M_4 (UHS POP)	Thiamethoxam 25% Wg 0.20 ml/l followed by 1% groundnut oil 10 ml/l	Neam leaf extract 1500 ppm 2 ml/l	Acephate 75 % SP 1.0 gm/l followed by spray 1%Neem oil 10 ml/l	Neam Leaf extract 1500 ppm 2 ml/l	Dimethoate 30 % EC 1.7ml/l followed by 1% groundnut oil 10 ml/l	Neam leaf extract 1500 ppm 2 ml/l

*DAT: Day After Transplanting, ** Zn-3.0%, Fe-2.0%, Mn-1.0%, B-0.50%, Mg-2.0%

Table S5. Status of PRSD in major papaya growing districts of Karnataka, India

Sl. No.	Taluk	Village	Name of Variety	Area (Acre)	Crop Stage (Months)	Previous crop	Surrounding Crop	PRSV Disease Incidence (%)	PRSV symptoms in field	Pathogenicity test	RT-PCR based detection of PRSV
**I**	**District : Bagalkote**										
1.	Bagalkote	Kolakachi	Ice Berg	1.00	04	Sugarcane	Tomato	0.7	GM	+ve	+ve
2.		Tulsigeri	RedLady	1.50	16	Brinjal	Pomegranate	100.0	GM, LC, SS, YM, Mo, LP, BF, LD, OS, S, RS, CS, DF	+ve	+ve
3.	Bilgi	Honakatti	RedLady	2.33	06	Brinjal	Sugarcane	100.0	GM, B, LC, SS, YM, Mo, LP, LD, OS	+ve	+ve
4.		Bilgi	RedLady	2.00	16	Sugarcane	Sugarcane	100.0	GM, LC, SS, YM, Mo, LP, BF, LD, OS, S, RS, CS, DF	+ve	+ve
5.	Hungund	Bannihatti	RedLady	1.08	09	Tomato	Maize	100.0	GM, B, LC, SS, Mo, LP, BF, LD, OS, S, RS	+ve	+ve
6.		Hiremagi	RedLady	2.00	10	Maize	Chilli	91.7	GM, B, LC, SS, YM, Mo, LP, BF, LD, OS, S	+ve	+ve
7.		Hiremagi	RedLady	1.42	12	Maize	Brinjal	94.1	GM, B, LC, ST, Mo, LP, BF, LD, OS, S, RS, CS, DF	+ve	+ve
8.		Hiremagi	RedLady	1.25	09	Tomato	Pomegranate	100.0	GM, B, LC, SS, YM, Mo, LP, BF, LD, OS, S, RS, CS, DF	+ve	+ve
							**Average**	**85.8**			
**II**	**District : Ballari**										
9.	Ballari	Belagal	RedLady	1.25	12	Papaya	Sapota	6.7	GM, B, LC, Mo	+ve	+ve
10.	Hagari bomanahalli	Kannehalli	RedLady	1.00	13	Maize	Banana	8.3	GM, B, LC, Mo	+ve	+ve
11.		Gaddikeri	RedLady	1.83	07	Maize	Sapota	100.0	GM, B, LC, SS, Mo, LP, BF, LD, OS, S	+ve	+ve
12.		Mailara	RedLady	1.42	08	Banana	Banana	47.1	GM, LC, SS, YM, Mo, LP, B, OS, S	+ve	+ve
13.	Sndur	Yelubenchi	Ice Berg	1.83	9	Maize	Banana	100.0	GM, B, LC, ST, Mo, LP, BF, LD, OS, S, RS	+ve	+ve
14.		Bilakudi	RedLady	2.50	10	Maize	Pomegranate	100.0	GM, B, LC, ST, YM, Mo, LP, BF, LD, OS, S, RS, CS, DF	+ve	+ve
15.		Basavanadurga	RedLady	1.00	08	Maize	Guava	100.0	GM, B, LC, ST, Mo, LP, BF, LD, OS, S,	+ve	+ve
16.		Vysapura	RedLady	0.92	11	Maize	Banana	100.0	GM, B, LC, SS, YM, Mo, LP, BF, LD, OS, S, RS	+ve	+ve
							**Average**	**70.3**			
**III**	**District : Belagavi**										
17.	Gokak	Bangwhad	RedLady	0.83	24	Capsicum	Chilli	100.0	GM, B, LC, SS, YM, Mo, LP, BF, LD, OS, S, RS, CS, DF	+ve	+ve
18.		Bangwhad	RedLady	1.25	17	Maize	Turmeric	100.0	GM, B, LC, SS, YM, Mo, LP, BF, LD, OS, S, RS, CS, DF	+ve	+ve
19.		Bangwhad	RedLady	1.00	05	Marigold	Sugarcane	8.3	GM	+ve	+ve
20.		Ghataprabha	RedLady	1.25	17	Tomato	Turmeric	80.0	GM, B, LC, SS, YM, Mo, LP, BF, OS, S, RS, CS, DF	+ve	+ve
21.		Ghataprabha	RedLady	1.25	04	Marigold	Sugarcane	5.2	GM	+ve	+ve
22.	Ramadurga	Mudakavi	RedLady	1.00	24	Sugarcane	Sugarcane	100.0	GM, B, LC, SS, YM, Mo, LP, BF, LD, OS, S, RS, CS, DF	+ve	+ve
23.		Vasavinagar	RedLady	1.67	24	Maize	Sugarcane	100.0	GM, B, LC, SS, YM, Mo, LP, BF, LD, OS, S, RS, CS, DF	+ve	+ve
24.		Vasavinagar	RedLady	1.25	24	Maize	Sugarcane	100.0	GM, B, LC, SS, YM, Mo, LP, BF, LD, OS, S, RS, CS, DF	+ve	+ve
25.		Thornur	RedLady	0.83	09	Groundnut	Sugarcane	100.0	GM, B, LC, SS, Mo, LP, BF, LD, OS, S, RS	+ve	+ve

26.		Thornur	RedLady	1.50	09	Cotton	Sugarcane	100.0	GM, B, LC, SS, YM, Mo, LP, B, LD, OS, S, RS	+ve	+ve
27.		Benakhati	RedLady	3.75	05	Sugarcane	Sugarcane	0.4	GM	+ve	+ve
28.	Savadatti	Kadavi Shivapura	RedLady	0.42	17	Brinjal	Banana	100.0	GM, B, LC, SS, YM, Mo, LP, BF, LD, OS, S, RS, CS, DF	+ve	+ve
29.		Yargheri	RedLady	0.83	17	Maize	Sugarcane	100.0	GM, B, LC, SS, YM, Mo, LP, BF, LD, OS, S, RS, CS, DF	+ve	+ve
30.		Yargheri	RedLady	0.67	17	Maize	Sugarcane	100.0	GM, B, LC, SS, YM, Mo, LP, BF, LD, OS, S, RS, CS, DF	+ve	+ve
31.	Savadatti	Yargheri	RedLady	0.75	17	Maize	Sugarcane	100.0	GM, LC, SS, YM, Mo, LP, B, LD, OS, S, RS, CS, DF	+ve	+ve
32.		Yargheri	RedLady	1.67	17	Maize	Sugarcane	100.0	GM, B, LC, SS, YM, Mo, LP, BF, LD, OS, S, RS, CS, DF	+ve	+ve
							**Average**	**80.8**			
**IV**	**District : Chitradurga**										
33.		Renukapura	RedLady	1.08	05	Chilli	Pomegranate	76.9	GM, LC, SS, Mo, LP, BF, OS, S	+ve	+ve
34.		Ydalagatte	RedLady	1.25	03	Tomato	Pomegranate	1.5	GM	+ve	+ve
35.		Katandevarakote	RedLady	1.00	02	Groundnut	Pomegranate	83.3	GM, LC, SS, Mo	+ve	+ve
36.		Renukapura	RedLady	1.67	06	Groundnut	Pomegranate	100.0	GM, LC, SS, YM, Mo, LP, B, LD, OS, S, RS, CS, DF	+ve	+ve
37.	Challakere	Katandevarakote	Ice Berg	1.00	05	Groundnut	Pomegranate	100.0	GM, LC, SS, YM, Mo, LP, LD, OS, S, RS, CS, DF	+ve	+ve
38.		Katandevarakot	RedLady	0.83	05	Ridge Gourd	Pomegranate	8.0	GM	+ve	+ve
39.		Dhoderri	RedLady	1.25	07	Tomato	Pomegranate	100.0	GM, B, LC, SS, YM, Mo, LP, BF, LD, OS, S	+ve	+ve
40.		Bharamasagara	RedLady	1.25	06	Groundnut	Pomegranate	80.0	GM, LC, SS, Mo, LP, B, OS, S	+ve	+ve
41.		Bharamasagara	RedLady	1.08	06	Groundnut	Pomegranate	7.7	GM	+ve	+ve
							**Average**	**61.9**			
**V**	**District : Gadag**										
42.		Harthi	RedLady	2.00	07	Banana	Banana	1.6	GM, LC	+ve	+ve
43.	Gadag	Hollalapura	RedLady	2.50	14	Cotton	Maize	100.0	GM, B, LC, SS, YM, Mo, LP, BF, LD, OS, S, RS, CS, DF	+ve	+ve
44.		Kiratageri	RedLady	1.25	17	Chickpea	Banana	100.0	GM, B, LC, SS, YM, Mo, LP, BF, LD, OS, S, RS, CS, DF	+ve	+ve
45.		Harthi	RedLady	1.92	08	Banana	Banana	0.4	GM	+ve	+ve
							**Average**	**50.5**			
**VI**	**District : Haveri**										
46.		Basapura	RedLady	1.42	07	Maize	Chilli	100.0	GM, B, LC, SS, YM, Mo, LP, BF, LD, OS, S	+ve	+ve
47.		Basapura	RedLady	1.00	08	Brinjal	Chilli	66.7	GM, B, LC, SS, Mo, LP, BF, OS, S, RS	+ve	+ve
48.		Jangamanakoppa	RedLady	2.42	12	Chilli	Maize	96.6	GM, B, LC, SS, YM, Mo, LP, BF, LD, OS, S, RS, CS, DF	+ve	+ve
49.	Haveri	Kancharagatti	RedLady	1.33	09	Maize	Maize	93.8	GM, B, LC, SS, YM, Mo, LP, BF, LD, OS, S, CS, RS	+ve	+ve
50.		Kancharagatti	RedLady	1.50	14	Maize	Tomato	100.0	GM, B, LC, SS, YM, Mo, LP, BF, LD, OS, S, RS, CS, DF	+ve	+ve
51.		Bommanakatti	RedLady	1.00	08	Maize	Maize	83.3	GM, B, LC, SS, Mo, LP, BF, OS, S, RS,	+ve	+ve
52.		Bommanakatti	RedLady	1.17	10	Maize	Maize	100.0	GM, B, LC, SS, Mo, LP, BF, LD, OS, S, RS, CS, DF	+ve	+ve
							**Average**	**91.5**			
**VII**	**District : Kalaburagi**										
53.	Afzalpur	Anoor	RedLady	1.17	11	Watermelon	Sugarcane	100.0	GM, LC, SS, Mo, LP, BF, LD, OS, S, RS, CS, DF	+ve	+ve

54.		Anoor	Ice Berg	2.00	02	Watermelon	Grapes	0.8	GM	+ve	+ve
55.		Tavarkeda	RedLady	1.00	11	Sugarcane	Watermelon	100.0	GM, LC, SS, YM, Mo, LP, BF, LD, OS, S, RS	+ve	+ve
56.	Aland	Moga (K)	RedLady	3.17	08	Banana	Grapes	100.0	GM, LC, SS, Mo, LP, BF, LD, OS, S	+ve	+ve
57.	Kalaburagi	Mulkunda (K)	RedLady	1.08	02	Watermelon	Watermelon	0.4	GM	+ve	+ve
58.		Mulkunda (K).	RedLady	1.25	02	Watermelon	Sugarcane	0.5	GM	+ve	+ve
59.		Gobur	RedLady	1.42	05	Watermelon	Watermelon	100.0	GM, LC, SS, Mo, LP, B, LD, OS, S	+ve	+ve
60.		Farahatabad	RedLady	1.00	16	Banana	Watermelon	100.0	GM, LC, SS, YM, Mo, LP, B, LD, OS, S, RS, CS, DF	+ve	+ve
61.		Hadagalarathi	RedLady	0.67	03	Toor Dal	Watermelon	100.0	GM, LC, SS, Mo,	+ve	+ve
62.		Kalangraga	RedLady	1.08	04	Watermelon	Watermelon	100.0	GM, LC, SS, Mo, LP, B, LD, OS	+ve	+ve
63.		Hadagalarathi	RedLady	1.00	08	Watermelon	Maize	100.0	GM, LC, SS, YM, Mo, LP, BF, LD, OS, S, RS,	+ve	+ve
64.		Mulkunda (K)	RedLady	2.42	06	Watermelon	Maize	0.2	GM, LC	+ve	+ve
65.		Mulkunda (K)	RedLady	2.50	07	Watermelon	Watermelon	0.9	GM, LC	+ve	+ve
66.		Sirsangi	RedLady	1.50	02	Banana	Watermelon	0.2	GM, LC	+ve	+ve
							**Average**	**57.3**			
**VIII**	**District : Koppal**										
67.	Koppal	Achar Thimapura	RedLady	0.83	05	Pomegranate	Guava	100.0	GM, LC, SS, Mo, LP,, LD, OS, S,	+ve	+ve
68.		Halawarthi	RedLady	5.00	09	Banana	Mango	100.0	GM, LC, SS, YM, Mo, LP, BF, LD, OS, S, RS	+ve	+ve
69.		Achar Thimapura	RedLady	3.75	09	Banana	Banana	100.0	GM, LC, SS, YM, Mo, LP, BF, LD, OS, S, RS, CS, DF	+ve	+ve
70.		Methagal	RedLady	2.50	08	Pomegranate	Pomegranate	100.0	GM, LC, SS, Mo, LP, BF, LD, OS, S	+ve	+ve
							**Average**	**100.0**			
**IX**	**District : Vijayapura**										
71.	Muddebihal	Nidagundi	RedLady	2.83	15	Watermelon	Tomato	100.0	GM, LC, SS, YM, Mo, LP, BF, LD, OS, S, RS, CS, DF	+ve	+ve
72.		Vanahalli	RedLady	1.42	15	Watermelon	Banana	100.0	GM, LC, SS, YM, Mo, LP, BF, LD, OS, S, RS, CS, DF	+ve	+ve
73.		Kavadimatti	RedLady	1.67	08	Banana	Maize	0.9	GM	+ve	+ve
							**Average**	**67.0**			
**X**	**District: Yadgiri**										
74.	Surapura	Narayanpur	Ice Berg	3.17	09	Watermelon	Maize	100.0	GM, LC, SS, Mo, LP, BF, LD, OS, S, RS	+ve	+ve
							**Average**	**100.0**			

Note: B: Blistering, BF: Bumps on fruits, CS: Chlorotic spot on fruits, DF: Distorted Fruit, GM: Green Mosaic, LC: Leaf Curling, LD: Leaves distortion, LP: Leaves Puckering, Mo: Mottling, OS: Oily Streak, RS: Rings Spot, S: Stunted Growth, SS: Shoestring, and YM: Yellow Mosaic; * samples not collected due to no incidence of PRSV.

Table S6. Pairwise percent amino acid sequence and nucleotide sequence identity between the PRSV isolated from papaya with other selected PRSV isolates

Sl. No	Potyvirus	Accession No.	Percentage amino acid sequence identity (%)	Percentage nucleotide sequence identity (%)
1.	PRSV-[IN:VC:Pap:15]	MF405299	**96.5**	**95.5**
2.	PRSV-[IN:TS:Hyd:HYD:Pap:15]	KP743981	**96.2**	**95.8**
3.	PRSV-[IN:MH:PM-H:Pap:15]	MF405295	94.8	93.1
4.	PRSV-[IN:MH:PS3-H:Pap:15]	MF405297	94.7	93.9
5.	PRSV-[IN:MH:PM-I:Pap:15]	MF405296	94.3	92.3
6.	PRSV-[IN:MH:PS3-H:Pap:15]	MF405298	93.7	91.2
7.	PRSV-[IN:ND:DEL:Pap:06]	EF017707	91.8	89
8.	PRSV-[PNG:8A:Pap:16]	MH404259	90.6	84.6
9.	PRSV-[PNG:12B:Pap:16]	MH404260	90.6	84.9
10.	PRSV-[PNG:16A:Pap:16]	MH404261	90.6	85
11.	PRSV-[PNG:18B:Pap:16]	MH404263	90.5	84.8
12.	PRSV-[TH:Ver:PDO:Mex-VrPO:Pap:13]	AY231130	90.4	85.3
13.	PRSV-[PNG:17B:Pap:16]	MH404262	90.4	84.7
14.	PRSV-[PNG:22A:Pap:16]	MH404264	90.4	84.5
15.	PRSV-[TW:TXG:Pap:18]	NC_001785	90.2	85.3
16.	PRSV-[US:Hi:HA:Pap:93]	S46722	90.2	85.3
17.	PRSV-[TW:TXG:Pap:05]	X67673	90.2	85.3
18.	PRSV-[US:PRSV-PTX:Pap:14]	KY271954	90.1	85
19.	PRSV-[US:HI:P/mutantHA5-1:Pap:84]	MT470188	90.1	85.2
20.	PRSV-[US:HI:PG:Pap:07]	EU126128	90.0	85
21.	PRSV-[CO:CH:PRSV_CH:Pap:14]	KT275938	90.0	84.6
22.	PRSV-[CO:VDR:PRSV_VR:Pap:14]	KT275937	89.9	84.6
23.	PRSV-[EC:Bab-Ec:Pap:17]	MH974109	89.8	84.7
24.	PRSV-[MX:PRSV.PC-VA:Pap:14]	MN203183	89.8	83.9
25.	PRSV-[MX:PRSV.CD-VA:Pap:14]	MN203186	89.7	84.6
26.	PRSV-[EC:PRSV_Pap_Ec:Pap:18]	MH974110	89.7	83.8
27.	PRSV-[MX:PRSV.PC-PS:Pap:14]	MN203185	89.7	83.7
28.	PRSV-[MX:PRSV.CD-PS:Pap:14]	MN203187	89.6	83.7
29.	PRSV-[MX:PRSV.PC-OS:Pap:14]	MN203184	89.6	83.6
30.	PRSV-[EC:Mild_Los_Rios:Pap:18]	MT747167	89.5	84.7

31. PRSV-[TH:Pap:02]	AY162218	89.2	80.5
32. PRSV-[VN:TG:PRSV-TG5:Pap:15]	MT470189	89.0	80.4
33. PRSV-[TW:pfT_3-AX-D-Ph:Pap:12]	JX448372	88.9	80.8
34. PRSV-[CN:Pap:16]	KY933061	88.9	80.8
35. PRSV-[TW:prT_3-AX-M-Ph:Pap:12]	JX448371	88.9	77.9
36. PRSV-[TW:YK:Pap:08]	X97251	88.8	80.7
37. PRSV-[TW:Ping-tong:Pap:06]	DQ340769	88.8	80.4
38. PRSV-[CN:HN-1:Pap:10]	HQ424465	88.8	80.8
39. PRSV-[CN:Hai:Haikou:PRSV-HNVb:Pap:13]	KF791028	88.8	80.2
40. PRSV-[TW:Ping-tong:Pap:05]	DQ340771	88.8	79.6
41. PRSV-[TH:Sam:SMK:Pap:02]	MT470190	88.8	80.4
42. PRSV-[TW:Ping-tong:Pap:05]	DQ340770	88.8	80.7
43. PRSV-[CN:Hai:Pap:11]	KF734962	88.7	80.7
44. PRSV-[TW:pFT_3-NP:Pap:12]	JX448373	88.7	79.6
45. PRSV-[TW:prT_3-AX-N-Ph:Pap:12]	JX448370	88.6	80.2
46. PRSV-[CN:Hai:Dongfang:HN-DF:Pap:12]	KT895257	88.6	80.2
47. PRSV-[TW:prT_3-AX-N-Ad6:Pap:12]	JX448369	88.6	80.2
48. PRSV-[CN:Hai:Lin:PRSV-LM:Pap:15]	KT633943	88.5	80.7
49. PRSV-[TW:P-5-19:Pap:07]	EU882728	88.5	80.3
50. PRSV-[IN:PRSVR3:Pap:13]	KJ755852	88.5	79.6
51. PRSV-[CN:P:Pap:06]	EF183499	88.4	79.8
52. PRSV-[IN:MH:Pun:Pap:15]	MH311882	87.0	81.2
53. PRSV-[PK:PK:Pap:15]	MT090406	86.7	79
54. PRSV-[IN:Meg:Umi:Pap:15]	MF356497	86.4	79.8
55. PRSV-[BD:BD-2:Pap:16]	MH397222	86.4	78.6
56. PRSV-[IN:WB:Pap:17]	LC482263	85.9	78.7
57. PRSV-[IN:HP:Pal:Pap:18]	MW030522	85.7	78.4
58. PRSV-[BD:BD-1:Pap:16]	MH444652	83.1	70.2

Table S7. Effect of insecticides and biorationals on PRSD incidence (during 2019-2020)

Treatment	Disease incidence (%) at											
	30 DAT*	60 DAT	90 DAT	120 DAT	150 DAT	180 DAT	210 DAT	240 DAT	270 DAT	300 DAT	330 DAT	360 DAT
T_1	0.00 (0.00)**	0.00 (0.00)	0.00 (0.00)	0.00 (0.00)	0.00 (0.00)	0.00 (0.00)	0.00 (0.00)	2.78 (5.59)	22.22 (28.03)	50.00 (45.05)	80.56 (68.57)	100 (90.00)
T_2	0.00 (0.00)	0.00 (0.00)	0.00 (0.00)	0.00 (0.00)	0.00 (0.00)	16.67 (23.62)	50.00 (45.05)	52.78 (46.80)	77.78 (66.49)	91.67 (76.38)	100 (90.00)	100 (90.00)
T_3	0.00 (0.00)	0.00 (0.00)	0.00 (0.00)	0.00 (0.00)	0.00 (0.00)	8.33 (13.62)	41.67 (39.63)	44.44 (41.60)	72.22 (63.40)	88.89 (73.94)	100 (90.00)	100 (90.00)
T_4	0.00 (0.00)	0.00 (0.00)	0.00 (0.00)	0.00 (0.00)	0.00 (0.00)	0.00 (0.00)	13.89 (18.03)	16.67 (23.62)	41.67 (39.79)	77.78 (62.65)	100 (90.00)	100 (90.00)
T_5	0.00 (0.00)	0.00 (0.00)	0.00 (0.00)	0.00 (0.00)	0.00 (0.00)	0.00 (0.00)	8.33 (13.62)	11.11 (19.22)	36.11 (35.59)	69.44 (56.81)	100 (90.00)	100 (90.00)
T_6	0.00 (0.00)	0.00 (0.00)	0.00 (0.00)	0.00 (0.00)	0.00 (0.00)	27.78 (31.75)	44.44 (41.80)	47.22 (43.40)	72.22 (58.46)	88.89 (74.41)	100 (90.00)	100 (90.00)
T_7	0.00 (0.00)	0.00 (0.00)	0.00 (0.00)	0.00 (0.00)	0.00 (0.00)	2.78 (5.59)	22.22 (27.03)	25.00 (29.46)	50.00 (45.37)	72.22 (58.57)	100 (90.00)	100 (90.00)
T_8	0.00 (0.00)	0.00 (0.00)	0.00 (0.00)	11.11 (16.06)	25.00 (29.79)	50.00 (45.16)	80.56 (68.25)	83.33 (70.21)	100 (90.00)	100 (90.00)	100 (90.00)	100 (90.00)
T_9	0.00 (0.00)	0.00 (0.00)	0.00 (0.00)	63.89 (53.25)	94.44 (78.81)	100 (90.00)	100 (90.00)	100 (90.00)	100 (90.00)	100 (90.00)	100 (90.00)	100 (90.00)
S Em ±	NS	0.00	0.00	3.08	2.15	3.88	6.91	5.41	8.7	5.66	3.89	NS
C D @ 5 %	NS	0.00	0.00	9.23	6.44	11.64	20.72	16.20	26.2	16.97	11.67	NS

Treatments	1st Spray at 30 DAT	2nd Spray at 60 DAT	3rd Spray at 90 DAT	4th Spray at 120 DAT	5th Spray at 150 DAT	6th Spray at 180 DAT	7th Spray at 210 DAT	8th Spray at 240 DAT
T_1	Tolfenpyrad 15% EC 1 ml/l →micronutrients***	Imidacloprid 17.8% SL 0.2 ml/l →micronutrients	Thiacloprid 21.7 SC 1 ml/l →micronutrients	Dinotefuran 20 % SG 0.5g/l →micronutrients	Tolfenpyrad 15% EC 1 ml/l →micronutrients	Imidacloprid 17.8% SL 0.2 ml/l →micronutrients	Thiacloprid 21.7 SC 1 ml/l →micronutrients	Dinotefuran 20 % SG 0.5g/l →micronutrients
T_2	1% Neem oil 10 ml/l →micronutrients	Pongamia oil 10 ml/l →micronutrients	Groundnut oil 10 ml/l →micronutrients	Mineral oil 10 ml/l →micronutrients	1% Neem oil 10 ml/l →micronutrients	Pongamia oil 10 ml/l →micronutrients	Groundnut oil 10 ml/l →micronutrients	Mineral oil 10 ml/l →micronutrients
T_3	Seaweed extract 4ml/l →micronutrient	Seaweed extract 4ml/l →micronutrient	Seaweed extract 4ml/l →micronutrient	Seaweed extract 4ml/l →micronutrient	Seaweed extract 4ml/l →micronutrient	Seaweed extract 4ml/l →micronutrient	Seaweed extract 4ml/l →micronutrient	Seaweed extract 4ml/l →micronutrient
T_4	Tolfenpyrad 15% EC 1 ml/l →micronutrients	1% Neem oil 10 ml/l →micronutrients	Imidacloprid 17.8% SL 0.2 ml/l →micronutrients	Pongamia oil 10 ml/l →micronutrients	Thiacloprid 21.7 SC 1ml/l →micronutrients	Groundnut oil 10 ml/l →micronutrients	Dinotefuran 20 % SG 0.5g/l →micronutrients	Mineral oil 10 ml/l →micronutrients
T_5	Tolfenpyrad 15% EC 1 ml/l →micronutrients	Seaweed extract 4ml/l →micronutrients	Imidacloprid 17.8% SL 0.2 ml/l →micronutrients	Seaweed extract 4ml/l →micronutrients	Thiacloprid 21.7 SC 1 ml/l →micronutrients	Seaweed extract 4ml/l →micronutrients	Dinotefuran 20 % SG 0.5g/l →micronutrients	Seaweed extract 4ml/l →micronutrients
T_6	1% Neem oil 10 ml/l →micronutrients	Seaweed extract 4ml/l →micronutrients	Pongamia oil 10 ml/l →micronutrients	Seaweed extract 1gm/l →micronutrients	Groundnut oil 10 ml/l →micronutrients	Seaweed extract 4ml/l →micronutrients	Mineral oil 10 ml/l →micronutrients	Seaweed extract 4ml/l →micronutrients
T_7	Tolfenpyrad 15% EC 1 ml/l →micronutrients	1%Neem oil 10 ml/l →micronutrients	Seaweed extract 4ml/l →micronutrients	Seaweed extract 4ml/l →micronutrients	Imidacloprid 17.8% SL 0.2 ml/l →micronutrients	Groundnut oil 10 ml/l →micronutrients	Seaweed extract 4ml/l →micronutrients	Seaweed extract 4ml/l →micronutrients
T_8	Micronutrients	Micronutrients	Micronutrients	Micronutrients	Micronutrients	Micronutrients	Micronutrients	Micronutrients
T_9	Control							

*DAT-Days After Transplanting, ** Figures in parentheses are arc sine transformed values, *** Zn-3.0%, Fe-2.0%, Mn-1.0%, B-0.50%, Mg-2.0%, → Followed by

Table S8. Effect of insecticides and biorationals on growth, yield and yield parameters of papaya influenced by PRSD (during 2019-2020)

Treatment	Plant height (cm) at 270 DAT*	Internodal length (cm) at 270 DAT	Plant girth (cm) at 270 DAT	No. of leaves per plants at 270 DAT	No. of Days taken for first flowering	No. of flowers at 270 DAT	No. of days taken from flowering to first fruit set	No. of days taken to first fruit set to harvest	No. of fruits per plant	Fruit diameter (cm)	Fruit length (cm)	Fruit breadth (cm)	Fruit cavity diameter (cm)	Fruit yield per plant (Kg)	Fruit yield per hectare (t)
T_1	225.66	4.09	40.37	29.41	92.89	72.77	93.47	125.06	25.97	12.73	27.36	14.31	6.75	57.92	178.74
T_2	204.72	3.60	37.36	23.11	105.44	31.77	103.36	109.86	16.06	9.49	19.83	9.81	6.01	26.49	81.75
T_3	206.16	3.72	38.22	27.14	100.69	57.97	100.53	118.03	23.11	11.31	23.83	11.23	6.45	48.53	149.77
T_4	213.91	4.09	39.55	28.25	99.08	56.41	95.39	123.25	22.00	11.15	23.61	11.89	6.50	42.48	131.09
T_5	216.44	4.09	39.66	28.57	96.03	59.61	95.83	123.28	24.17	12.39	24.67	12.49	6.64	51.73	159.62
T_6	205.52	3.48	37.69	26.27	101.75	54.11	100.31	113.83	21.64	10.48	22.33	11.35	6.33	40.38	124.60
T_7	214.57	3.71	39.05	27.23	100.03	56.38	100.89	112.72	21.25	11.10	23.36	11.48	6.35	41.33	127.55
T_8	160.69	3.39	33.97	20.46	105.36	20.44	110.61	97.75	5.67	8.48	17.36	9.34	5.88	6.38	19.67
T_9	135.27	3.27	20.83	19.72	106.69	14.86	114.64	98.00	1.33	8.13	14.64	8.79	5.56	0.83	2.57
S Em ±	3.05	0.07	0.29	0.33	0.21	1.10	0.72	0.34	0.86	0.13	0.25	0.14	0.08	1.04	3.21
C D @ 5 %	9.13	0.20	0.87	0.98	0.62	3.29	2.17	1.01	2.57	0.39	0.74	0.43	0.23	3.12	9.62

Treatments	1st Spray at 30 DAT	2nd Spray at 60 DAT	3rd Spray at 90 DAT	4th Spray at 120DAT	5th Spray at 150 DAT	6th Spray at 180 DAT	7th Spray at 210DAT	8th Spray at 240DAT
T_1	Tolfenpyrad 15% EC 1 ml/l →micronutrients***	Imidacloprid 17.8% SL 0.2 ml/l →micronutrients	Thiacloprid 21.7 SC 1 ml/l →micronutrients	Dinotefuran 20 % SG 0.5g/l →micronutrients	Tolfenpyrad 15% EC 1 ml/l →micronutrients	Imidacloprid 17.8% SL 0.2 ml/l →micronutrients	Thiacloprid 21.7 SC 1 ml/l →micronutrients	Dinotefuran 20 % SG 0.5g/l →micronutrients
T_2	1% Neem oil 10 ml/l →micronutrients	Pongamia oil 10 ml/l →micronutrients	Groundnut oil 10 ml/l →micronutrients	Mineral oil 10 ml/l →micronutrients	1% Neem oil 10 ml/l →micronutrients	Pongamia oil 10 ml/l →micronutrients	Groundnut oil 10 ml/l →micronutrients	Mineral oil 10 ml/l →micronutrients
T_3	Seaweed extract 4ml/l →micronutrient	Seaweed extract 4ml/l →micronutrient	Seaweed extract 4ml/l →micronutrient	Seaweed extract 4ml/l →micronutrient	Seaweed extract 4ml/l →micronutrient	Seaweed extract 4ml/l →micronutrient	Seaweed extract 4ml/l →micronutrient	Seaweed extract 4ml/l →micronutrient
T_4	Tolfenpyrad 15% EC 1 ml/l →micronutrients	1% Neem oil 10 ml/l →micronutrients	Imidacloprid 17.8% SL 0.2 ml/l →micronutrients	Pongamia oil 10 ml/l →micronutrients	Thiacloprid 21.7 SC 1ml/l →micronutrients	Groundnut oil 10 ml/l →micronutrients	Dinotefuran 20 % SG 0.5g/l →micronutrients	Mineral oil 10 ml/l →micronutrients
T_5	Tolfenpyrad 15% EC 1 ml/l →micronutrients	Seaweed extract 4ml/l →micronutrients	Imidacloprid 17.8% SL 0.2 ml/l →micronutrients	Seaweed extract 4ml/l →micronutrients	Thiacloprid 21.7 SC 1 ml/l →micronutrients	Seaweed extract 4ml/l →micronutrients	Dinotefuran 20 % SG 0.5g/l →micronutrients	Seaweed extract 4ml/l →micronutrients
T_6	1% Neem oil 10 ml/l →micronutrients	Seaweed extract 4ml/l →micronutrients	Pongamia oil 10 ml/l →micronutrients	Seaweed extract 1gm/l →micronutrients	Groundnut oil 10 ml/l →micronutrients	Seaweed extract 4ml/l →micronutrients	Mineral oil 10 ml/l →micronutrients	Seaweed extract 4ml/l →micronutrients
T_7	Tolfenpyrad 15% EC 1 ml/l →micronutrients	1%Neem oil 10 ml/l →micronutrients	Seaweed extract 4ml/l →micronutrients	Seaweed extract 4ml/l →micronutrients	Imidacloprid 17.8% SL 0.2 ml/l →micronutrients	Groundnut oil 10 ml/l →micronutrients	Seaweed extract 4ml/l →micronutrients	Seaweed extract 4ml/l →micronutrients
T_8	Micronutrients	Micronutrients	Micronutrients	Micronutrients	Micronutrients	Micronutrients	Micronutrients	Micronutrients
T_9	Control							

*DAT-Days After Transplanting, ** Zn-3.0%, Fe-2.0%, Mn-1.0%, B-0.50%, Mg-2.0%, →Followed by

Table S9. Effect of insecticides and biorationals on PRSD incidence (during 2020-2021)

Treatment	Disease incidence (%) at											
	30 DAT*	60 DAT	90 DAT	120 DAT	150 DAT	180 DAT	210 DAT	240 DAT	270 DAT	300 DAT	330 DAT	360 DAT
T_1	0.00 (0.00)**	0.00 (0.00)	0.00 (0.00)	0.00 (0.00)	0.00 (0.00)	0.00 (0.00)	2.97 (9.50)	7.61 (15.98)	25.92 30.59)	51.81 (46.13)	87.94 (73.22)	100 (90.00)
T_2	0.00 (0.00)	0.00 (0.00)	0.00 (0.00)	0.00 (0.00)	8.00 (16.42)	22.64 (28.35)	58.31 (49.83)	70.33 (57.06)	81.00 (68.49)	93.97 (81.61)	100 (90.00)	100 (90.00)
T_3	0.00 (0.00)	0.00 (0.00)	0.00 (0.00)	0.00 (0.00)	0.00 (0.00)	10.83 (18.84)	44.42 (41.60)	51.14 (45.66)	75.92 (65.59)	89.78 (74.63)	100 (90.00)	100 (90.00)
T_4	0.00 (0.00)	0.00 (0.00)	0.00 (0.00)	0.00 (0.00)	0.00 (0.00)	5.22 (10.83)	16.86 (23.80)	22.42 (28.18)	50.89 (45.88)	85.36 (67.71)	100 (90.00)	100 (90.00)
T_5	0.00 (0.00)	0.00 (0.00)	0.00 (0.00)	0.00 (0.00)	0.00 (0.00)	0.00 (0.00)	13.61 (21.49)	16.39 (23.88)	43.50 (41.15)	74.31 (59.86)	100 (90.00)	100 (90.00)
T_6	0.00 (0.00)	0.00 (0.00)	0.00 (0.00)	0.00 (0.00)	7.31 (15.66)	22.42 (28.23)	47.64 (43.64)	50.67 (45.38)	76.36 (61.12)	92.11 (80.30)	100 (90.00)	100 (90.00)
T_7	0.00 (0.00)	0.00 (0.00)	0.00 (0.00)	0.00 (0.00)	0.00 (0.00)	1.61 (5.49)	24.97 (29.16)	31.44 (33.95)	54.61 (48.25)	78.89 (62.73)	100 (90.00)	100 (90.00)
T_8	0.00 (0.00)	0.00 (0.00)	0.00 (0.00)	10.14 (18.32)	26.11 (30.57)	61.11 (51.60)	82.39 (69.44)	90.72 (79.39)	100 (90.00)	100 (90.00)	100 (90.00)	100 (90.00)
T_9	0.00 (0.00)	0.00 (0.00)	1.14 (4.98)	67.33 (55.14)	86.31 (68.71)	100 (90.00)	100 (90.00)	100 (90.00)	100 (90.00)	100 (90.00)	100 (90.00)	100 (90.00)
S Em ±	0.00	0.00	0.84	0.78	1.61	2.76	4.88	3.68	7.35	5.19	2.80	0.00
C D @ 5 %	0.00	0.00	2.53	2.33	4.83	8.26	14.63	11.04	22.04	15.56	8.38	0.00

Treatments	1st Spray at 30 DAT	2nd Spray at 60 DAT	3rd Spray at 90 DAT	4th Spray at 120 DAT	5th Spray at 150 DAT	6th Spray at 180 DAT	7th Spray at 210 DAT	8th Spray at 240 DAT
T_1	Tolfenpyrad 15% EC 1 ml/l →micronutrients***	Imidacloprid 17.8% SL 0.2 ml/l →micronutrients	Thiacloprid 21.7 SC 1 ml/l →micronutrients	Dinotefuran 20 % SG 0.5g/l →micronutrients	Tolfenpyrad 15% EC 1 ml/l →micronutrients	Imidacloprid 17.8% SL 0.2 ml/l →micronutrients	Thiacloprid 21.7 SC 1 ml/l →micronutrients	Dinotefuran 20 % SG 0.5g/l →micronutrients
T_2	1% Neem oil 10 ml/l →micronutrients	Pongamia oil 10 ml/l →micronutrients	Groundnut oil 10 ml/l →micronutrients	Mineral oil 10 ml/l →micronutrients	1% Neem oil 10 ml/l →micronutrients	Pongamia oil 10 ml/l →micronutrients	Groundnut oil 10 ml/l →micronutrients	Mineral oil 10 ml/l →micronutrients
T_3	Seaweed extract 4ml/l →micronutrient	Seaweed extract 4ml/l →micronutrient	Seaweed extract 4ml/l →micronutrient	Seaweed extract 4ml/l →micronutrient	Seaweed extract 4ml/l →micronutrient	Seaweed extract 4ml/l →micronutrient	Seaweed extract 4ml/l →micronutrient	Seaweed extract 4ml/l →micronutrient
T_4	Tolfenpyrad 15% EC 1 ml/l →micronutrients	1% Neem oil 10 ml/l →micronutrients	Imidacloprid 17.8% SL 0.2 ml/l →micronutrients	Pongamia oil 10 ml/l →micronutrients	Thiacloprid 21.7 SC 1ml/l →micronutrients	Groundnut oil 10 ml/l →micronutrients	Dinotefuran 20 % SG 0.5g/l →micronutrients	Mineral oil 10 ml/l →micronutrients
T_5	Tolfenpyrad 15% EC 1 ml/l →micronutrients	Seaweed extract 4ml/l →micronutrients	Imidacloprid 17.8% SL 0.2 ml/l →micronutrients	Seaweed extract 4ml/l →micronutrients	Thiacloprid 21.7 SC 1 ml/l →micronutrients	Seaweed extract 4ml/l →micronutrients	Dinotefuran 20 % SG 0.5g/l →micronutrients	Seaweed extract 4ml/l →micronutrients
T_6	1% Neem oil 10 ml/l →micronutrients	Seaweed extract 4ml/l →micronutrients	Pongamia oil 10 ml/l →micronutrients	Seaweed extract 1gm/l →micronutrients	Groundnut oil 10 ml/l →micronutrients	Seaweed extract 4ml/l →micronutrients	Mineral oil 10 ml/l →micronutrients	Seaweed extract 4ml/l →micronutrients
T_7	Tolfenpyrad 15% EC 1 ml/l →micronutrients	1%Neem oil 10 ml/l →micronutrients	Seaweed extract 4ml/l →micronutrients	Seaweed extract 4ml/l →micronutrients	Imidacloprid 17.8% SL 0.2 ml/l →micronutrients	Groundnut oil 10 ml/l →micronutrients	Seaweed extract 4ml/l →micronutrients	Seaweed extract 4ml/l →micronutrients
T_8	Micronutrients	Micronutrients	Micronutrients	Micronutrients	Micronutrients	Micronutrients	Micronutrients	Micronutrients
T_9	Control							

*DAT-Days After Transplanting, ** Figures in parentheses are arc sine transformed values, *** Zn-3.0%, Fe-2.0%, Mn-1.0%, B-0.50%, Mg-2.0%, → Followed by

Table S10: Effect of insecticides and biorationals on growth, yield and yield parameters of papaya influenced by PRSD (during 2020-2021)

Treatment	Plant height (cm) at 270 DAT*	Internodal length (cm) at 270 DAT	Plant girth (cm) at 270 DAT	No. of leaves per plant at 270 DAT	No. of days taken for first flowering	No. of flowers at 270 DAT	No. of days taken from flowering to first fruit set	No. of days taken to first fruit set to harvest	No. of fruits per plant	Fruit diameter (cm)	Fruit length (cm)	Fruit breadth (cm)	Fruit cavity diameter (cm)	Fruit yield per plant (Kg)	Fruit yield per hectare (t)
T_1	226.19	4.13	40.53	29.58	93.14	73.35	94.56	126.22	26.21	13.53	28.19	14.56	6.88	57.81	178.39
T_2	205.24	3.61	37.52	23.27	105.22	32.33	104.44	111.03	16.39	10.29	20.67	10.05	6.14	25.87	79.83
T_3	206.69	3.70	38.30	27.25	100.97	58.55	101.61	118.86	22.94	11.54	25.67	11.48	6.62	43.13	133.11
T_4	214.44	4.10	39.41	28.33	99.53	56.99	96.47	123.75	22.86	12.95	25.78	12.47	6.74	42.42	130.92
T_5	216.97	4.11	39.71	28.77	95.86	60.16	96.92	124.47	24.26	13.18	26.17	12.61	6.82	52.60	162.33
T_6	206.05	3.49	37.80	26.43	102.11	54.69	101.39	115.00	21.73	11.16	23.53	10.73	6.48	41.15	127.00
T_7	214.41	3.75	39.19	27.39	100.36	56.96	101.97	119.22	22.26	11.33	25.42	11.64	6.56	42.16	130.12
T_8	161.22	3.40	34.13	20.62	105.58	21.02	111.69	98.92	5.65	9.27	18.19	9.59	6.01	6.48	20.01
T_9	135.79	3.28	21.07	19.88	106.92	15.44	115.72	99.17	1.05	8.93	15.47	9.03	5.69	0.79	2.45
S Em ±	2.59	0.06	0.27	0.33	0.30	1.11	0.72	0.34	0.82	0.11	0.20	0.09	0.08	1.06	3.28
C D @ 5 %	7.77	0.18	0.80	0.99	0.90	3.33	2.17	1.03	2.46	0.32	0.60	0.27	0.24	3.19	9.84

Treatments	1st Spray at 30 DAT	2nd Spray at 60 DAT	3rd Spray at 90 DAT	4th Spray at 120 DAT	5th Spray at 150 DAT	6th Spray at 180 DAT	7th Spray at 210 DAT	8th Spray at 240 DAT
T_1	Tolfenpyrad 15% EC 1 ml/l →micronutrients**	Imidacloprid 17.8% SL 0.2 ml/l →micronutrients	Thiacloprid 21.7 SC 1 ml/l →micronutrients	Dinotefuran 20 % SG 0.5g/l →micronutrients	Tolfenpyrad 15% EC 1 ml/l →micronutrients	Imidacloprid 17.8% SL 0.2 ml/l →micronutrients	Thiacloprid 21.7 SC 1 ml/l →micronutrients	Dinotefuran 20 % SG 0.5g/l →micronutrients
T_2	1% Neem oil 10 ml/l →micronutrients	Pongamia oil 10 ml/l →micronutrients	Groundnut oil 10 ml/l →micronutrients	Mineral oil 10 ml/l →micronutrients	1% Neem oil 10 ml/l →micronutrients	Pongamia oil 10 ml/l →micronutrients	Groundnut oil 10 ml/l →micronutrients	Mineral oil 10 ml/l →micronutrients
T_3	Seaweed extract 4ml/l →micronutrient	Seaweed extract 4ml/l →micronutrient	Seaweed extract 4ml/l →micronutrient	Seaweed extract 4ml/l →micronutrient	Seaweed extract 4ml/l →micronutrient	Seaweed extract 4ml/l →micronutrient	Seaweed extract 4ml/l →micronutrient	Seaweed extract 4ml/l →micronutrient
T_4	Tolfenpyrad 15% EC 1 ml/l →micronutrients	1% Neem oil 10 ml/l →micronutrients	Imidacloprid 17.8% SL 0.2 ml/l →micronutrients	Pongamia oil 10 ml/l →micronutrients	Thiacloprid 21.7 SC 1ml/l →micronutrients	Groundnut oil 10 ml/l →micronutrients	Dinotefuran 20 % SG 0.5g/l →micronutrients	Mineral oil 10 ml/l →micronutrients
T_5	Tolfenpyrad 15% EC 1 ml/l →micronutrients	Seaweed extract 4ml/l →micronutrients	Imidacloprid 17.8% SL 0.2 ml/l →micronutrients	Seaweed extract 4ml/l →micronutrients	Thiacloprid 21.7 SC 1 ml/l →micronutrients	Seaweed extract 4ml/l →micronutrients	Dinotefuran 20 % SG 0.5g/l →micronutrients	Seaweed extract 4ml/l →micronutrients
T_6	1% Neem oil 10 ml/l →micronutrients	Seaweed extract 4ml/l →micronutrients	Pongamia oil 10 ml/l →micronutrients	Seaweed extract 1gm/l →micronutrients	Groundnut oil 10 ml/l →micronutrients	Seaweed extract 4ml/l →micronutrients	Mineral oil 10 ml/l →micronutrients	Seaweed extract 4ml/l →micronutrients
T_7	Tolfenpyrad 15% EC 1 ml/l →micronutrients	1%Neem oil 10 ml/l →micronutrients	Seaweed extract 4ml/l →micronutrients	Seaweed extract 4ml/l →micronutrients	Imidacloprid 17.8% SL 0.2 ml/l →micronutrients	Groundnut oil 10 ml/l →micronutrients	Seaweed extract 4ml/l →micronutrients	Seaweed extract 4ml/l →micronutrients
T_8	Micronutrients	Micronutrients	Micronutrients	Micronutrients	Micronutrients	Micronutrients	Micronutrients	Micronutrients
T_9	Control							

*DAT-Days After Transplanting, ** Zn-3.0%, Fe-2.0%, Mn-1.0%, B-0.50%, Mg-2.0%, → Followed by

Table S11: Economics of the experiments on management of PRSD using insecticides and biorationals

Treatments	Yield (t/ha)	Gross returns (₹ /ha)*	Additional returns over control	Cost of cultivation (₹ /ha)	Cost of treatment (₹ /ha)	Total cost (₹ /ha)	Additional cost over control	Net returns (₹ /ha)	ICBR	B:C
T_1	178.56	10,71,360	10,56,300	2,93,536	8,760	3,02,296	8,760	7,69,064	121	3.54
T_2	80.79	4,84,740	4,69,680	2,93,536	8,038	3,01,574	8,038	1,83,166	58	1.61
T_3	141.44	8,48,640	8,33,580	2,93,536	18,350	3,11,886	18,350	5,36,754	45	2.72
T_4	131.01	7,86,060	7,71,000	2,93,536	8,767	3,02,303	8,767	4,83,757	88	2.60
T_5	160.97	9,65,820	9,50,760	2,93,536	14,055	3,07,591	14,055	6,58,229	68	3.14
T_6	125.8	7,54,800	7,39,740	2,93,536	12,598	3,06,134	12,598	4,48,666	59	2.47
T_7	128.83	7,72,980	7,57,920	2,93,536	13,835	3,07,371	13,835	4,65,609	55	2.51
T_8	19.84	1,19,040	1,03,980	2,93,536	2,350	2,95,886	2,350	-1,76,846	44	0.40
T_9	2.51	15,060	0	2,93,536	0	2,93,536	0	-2,78,476	0	0.05

Treatments	1st Spray at 30 DAT	2nd Spray at 60 DAT	3rd Spray at 90 DAT	4th Spray at 120 DAT	5th Spray at 150 DAT	6th Spray at 180 DAT	7th Spray at 210 DAT	8th Spray at 240 DAT
T_1	Tolfenpyrad 15% EC 1 ml/l →micronutrients**	Imidacloprid 17.8% SL 0.2 ml/l →micronutrients	Thiacloprid 21.7 SC 1 ml/l →micronutrients	Dinotefuran 20 % SG 0.5g/l →micronutrients	Tolfenpyrad 15% EC 1 ml/l →micronutrients	Imidacloprid 17.8% SL 0.2 ml/l →micronutrients	Thiacloprid 21.7 SC 1 ml/l →micronutrients	Dinotefuran 20 % SG 0.5g/l →micronutrients
T_2	1% Neem oil 10 ml/l →micronutrients	Pongamia oil 10 ml/l →micronutrients	Groundnut oil 10 ml/l →micronutrients	Mineral oil 10 ml/l →micronutrients	1% Neem oil 10 ml/l →micronutrients	Pongamia oil 10 ml/l →micronutrients	Groundnut oil 10 ml/l →micronutrients	Mineral oil 10 ml/l →micronutrients
T_3	Seaweed extract 4ml/l →micronutrient	Seaweed extract 4ml/l →micronutrient	Seaweed extract 4ml/l →micronutrient	Seaweed extract 4ml/l →micronutrient	Seaweed extract 4ml/l →micronutrient	Seaweed extract 4ml/l →micronutrient	Seaweed extract 4ml/l →micronutrient	Seaweed extract 4ml/l →micronutrient
T_4	Tolfenpyrad 15% EC 1 ml/l →micronutrients	1% Neem oil 10 ml/l →micronutrients	Imidacloprid 17.8% SL 0.2 ml/l →micronutrients	Pongamia oil 10 ml/l →micronutrients	Thiacloprid 21.7 SC 1ml/l →micronutrients	Groundnut oil 10 ml/l →micronutrients	Dinotefuran 20 % SG 0.5g/l →micronutrients	Mineral oil 10 ml/l →micronutrients
T_5	Tolfenpyrad 15% EC 1 ml/l →micronutrients	Seaweed extract 4ml/l →micronutrients	Imidacloprid 17.8% SL 0.2 ml/l →micronutrients	Seaweed extract 4ml/l →micronutrients	Thiacloprid 21.7 SC 1 ml/l →micronutrients	Seaweed extract 4ml/l →micronutrients	Dinotefuran 20 % SG 0.5g/l →micronutrients	Seaweed extract 4ml/l →micronutrients
T_6	1% Neem oil 10 ml/l →micronutrients	Seaweed extract 4ml/l →micronutrients	Pongamia oil 10 ml/l →micronutrients	Seaweed extract 1gm/l →micronutrients	Groundnut oil 10 ml/l →micronutrients	Seaweed extract 4ml/l →micronutrients	Mineral oil 10 ml/l →micronutrients	Seaweed extract 4ml/l →micronutrients
T_7	Tolfenpyrad 15% EC 1 ml/l →micronutrients	1%Neem oil 10 ml/l →micronutrients	Seaweed extract 4ml/l →micronutrients	Seaweed extract 4ml/l →micronutrients	Imidacloprid 17.8% SL 0.2 ml/l →micronutrients	Groundnut oil 10 ml/l →micronutrients	Seaweed extract 4ml/l →micronutrients	Seaweed extract 4ml/l →micronutrients
T_8	Micronutrients	Micronutrients	Micronutrients	Micronutrients	Micronutrients	Micronutrients	Micronutrients	Micronutrients
T_9	Control							

* Calculated using market value of papaya @ Rs.6/Kg, ** Zn-3.0%, Fe-2.0%, Mn-1.0%, B-0.50%, Mg-2.0%, → Followed by

Table S12: Effect of IDM modules on PRSD incidence at different crop growth stages

	Disease incidence (%) at										
**Module**	**30 DAT***	**60 DAT**	**90 DAT**	**120 DAT**	**150 DAT**	**180 DAT**	**210 DAT**	**240 DAT**	**270 DAT**	**300 DAT**	**330 DAT**
M_1	0.00	0.00	0.00	0.00	0.00	0.44	4.14	20.11	56.44	80.64	100
M_2	0.00	0.00	0.00	0.00	1.14	10.72	14.56	40.47	71.64	100	100
M_3	0.00	0.00	0.00	0.00	1.93	11.89	15.25	47.42	74.53	100	100
M_4 (UHS POP)	0.00	0.00	0.00	0.00	2.76	16.63	23.81	51.11	81.43	100	100

**Module**	**1st Spray at 20 DAT**	**2nd Spray at 40 DAT**	**3rd Spray at 60 DAT**	**4th Spray at 80 DAT**	**5th Spray at 100 DAT**	**6th Spray at 120 DAT**	**7th Spray at 140 DAT**	**8th Spray at 160 DAT**	**9th Spray at 180 DAT**	**10th Spray at 200 DAT**	**11th Spray at 220 DAT**	**12th Spray at 240 DAT**
M_1	Tolfenpyrad 15 EC 1 ml/l → micronutrients**	Imidacloprid 17.8% SL0.2ml/l → micronutrients	Thiacloprid 21.7 SC 1ml/l → micronutrients	Dinotefuran 20 % SG 0.5g/l →micronutrients	Tolfenpyrad 15EC 1 ml/l → micronutrients	Imidacloprid 17.8% SL 0.2 ml/l → micronutrients	Thiacloprid 21.7 SC 1 ml/l → micronutrients	Dinotefuran 20 % SG 0.5g/l → micronutrients	Tolfenpyrad 15EC 1 ml/l → micronutrients	Imidacloprid 17.8% SL 0.2 ml/l → micronutrients	Thiacloprid 21.7 SC 1 ml/l → micronutrients	Dinotefuran 20 % SG 0.5g/l → micronutrients
M_2	Tolfenpyrad 15EC 1 ml/l → micronutrients	Seaweed extract 4ml/l → micronutrients	Imidacloprid 17.8% SL 0.2 ml/l → micronutrients	Seaweed extract 4ml/l → micronutrients	Thiacloprid 21.7 SC 1 ml/l micronutrients	Seaweed extract 4ml/l → micronutrients	Dinotefuran 20 % SG 0.5g/l → micronutrients	Seaweed extract 4ml/l → micronutrients	Tolfenpyrad 15EC 1 ml/l → micronutrients	Seaweed extract 4ml/l → micronutrients	Imidacloprid 17.8% SL 0.2 ml/l → micronutrients	Seaweed extract 4ml/l → micronutrients
M_3	Tolfenpyrad 15EC 1 ml/l → micronutrients	Seaweed extract 4ml/l → micronutrients	Imidacloprid 17.8% SL 0.2 ml/l → micronutrients	Seaweed extract 4ml/l → micronutrients	Thiacloprid 21.7 SC 1 ml/l micronutrients	Seaweed extract 4ml/l → micronutrients	Dinotefuran 20 % SG 0.5g/l → micronutrients	Seaweed extract 4ml/l → micronutrients	1%Neem oil 10 ml/l → micronutrients	Seaweed extract 4ml/l → micronutrients	Groundnut oil 10 ml/l → micronutrients	Seaweed extract 4ml/l → micronutrients
M_4 (UHS POP)	Dimethoate 30 % EC 1.7ml/l-1% Neem oil 10 ml/l	Neam Leaf Extract 1500 ppm 2 ml/l	Oxydemeton Methyl 25% EC 1.5ml/l-1% Groundnut oil 10 ml/l	Neam Leaf Extract 1500 ppm 2 ml/l	Imidacloprid 17.8% SL 0.25ml/l-1%Neem oil 10 ml/l	Neam Leaf Extract 1500 ppm 2 ml/l	Thiamethoxam 25% Wg 0.20 ml/l-1% Groundnut oil 10 ml/l	Neam Leaf Extract 1500 ppm 2 ml/l	Acephate 75 % SP 1.0 gm/l-1%Neem oil 10 ml/l	Neam Leaf Extract 1500 ppm 2 ml/l	Dimethoate 30 % EC 1.7ml/l-1% Groundnut oil 10 ml/l	Neam Leaf Extract 1500 ppm 2 ml/l

*DAT-Days After Transplanting, ** Zn-3.0%, Fe-2.0%, Mn-1.0%, B-0.50%, Mg-2.0%, → Followed by

Table S13. Effect of IDM modules on growth, yield and yield parameters of papaya influenced by PRSD

Module	Plant height (cm) at 270 DAT*	Internodal length (cm) at 270 DAT	Plant girth (cm) at 270 DAT	No. of leaves per plants at 270 DAT	No. of days taken for first flowering	No. of flowers at 270 DAT	No. of days taken from flowering to first fruit set	No. of days taken to first fruit set to harvest	No. of fruits per plant	Fruit diameter (cm)	Fruit length (cm)	Fruit breadth (cm)	Fruit cavity diameter (cm)	Fruit yield per plant (Kg)	Fruit yield per hectare (t)
M_1	227.02	4.24	40.46	29.19	92.78	73.38	94.63	125.61	28.31	14.23	28.00	14.63	7.14	62.40	192.56
M_2	218.64	4.17	39.63	28.78	94.35	60.41	95.71	125.34	25.33	13.19	26.03	12.49	6.78	54.82	169.19
M_3	216.30	4.12	39.59	28.66	94.47	60.30	95.89	125.38	25.24	13.07	25.83	12.37	6.67	54.55	168.34
M_4 (UHS POP)	216.11	4.08	39.36	28.25	98.00	56.95	96.10	125.43	25.17	12.95	25.81	12.25	6.58	54.54	168.32

Module	1st Spray at 20 DAT	2nd Spray at 40 DAT	3rd Spray at 60 DAT	4th Spray at 80 DAT	5th Spray at 100 DAT	6th Spray at 120 DAT	7th Spray at 140 DAT	8th Spray at 160 DAT	9th Spray at 180 DAT	10th Spray at 200 DAT	11th Spray at 220 DAT	12th Spray at 240 DAT
M_1	Tolfenpyrad 15 EC 1 ml/l → micronutrients**	Imidacloprid 17.8% SL0.2ml/l → micronutrients	Thiacloprid 21.7 SC 1ml/l → micronutrients	Dinotefuran 20 % SG 0.5g/l →micronutrients	Tolfenpyrad 15EC 1 ml/l → micronutrients	Imidacloprid 17.8% SL 0.2 ml/l → micronutrients	Thiacloprid 21.7 SC 1 ml/l → micronutrients	Dinotefuran 20 % SG 0.5g/l → micronutrients	Tolfenpyrad 15EC 1 ml/l → micronutrients	Imidacloprid 17.8% SL 0.2 ml/l → micronutrients	Thiacloprid 21.7 SC 1 ml/l → micronutrients	Dinotefuran 20 % SG 0.5g/l → micronutrients
M_2	Tolfenpyrad 15EC 1 ml/l → micronutrients	Seaweed extract 4ml/l → micronutrients	Imidacloprid 17.8% SL 0.2 ml/l → micronutrients	Seaweed extract 4ml/l → micronutrients	Thiacloprid 21.7 SC 1 ml/l → micronutrients	Seaweed extract 4ml/l → micronutrients	Dinotefuran 20 % SG 0.5g/l → micronutrients	Seaweed extract 4ml/l → micronutrients	Tolfenpyrad 15EC 1 ml/l → micronutrients	Seaweed extract 4ml/l → micronutrients	Imidacloprid 17.8% SL 0.2 ml/l → micronutrients	Seaweed extract 4ml/l → micronutrients
M_3	Tolfenpyrad 15EC 1 ml/l → micronutrients	Seaweed extract 4ml/l → micronutrients	Imidacloprid 17.8% SL 0.2 ml/l → micronutrients	Seaweed extract 4ml/l → micronutrients	Thiacloprid 21.7 SC 1 ml/l → micronutrients	Seaweed extract 4ml/l → micronutrients	Dinotefuran 20 % SG 0.5g/l → micronutrients	Seaweed extract 4ml/l → micronutrients	1%Neem oil 10 ml/l → micronutrients	Seaweed extract 4ml/l → micronutrients	Groundnut oil 10 ml/l → micronutrients	Seaweed extract 4ml/l → micronutrients
M_4 (UHS POP)	Dimethoate 30 % EC 1.7ml/l-1% Neem oil 10 ml/l	Neam Leaf Extract 1500 ppm 2 ml/l	Oxydemeton Methyl 25% EC 1.5ml/l-1% Groundnut oil 10 ml/l	Neam Leaf Extract 1500 ppm 2 ml/l	Imidacloprid 17.8% SL 0.25ml/l-1%Neem oil 10 ml/l	Neam Leaf Extract 1500 ppm 2 ml/l	Thiamethoxam 25% Wg 0.20 ml/l-1% Groundnut oil 10 ml/l	Neam Leaf Extract 1500 ppm 2 ml/l	Acephate 75 % SP 1.0 gm/l-1%Neem oil 10 ml/l	Neam Leaf Extract 1500 ppm 2 ml/l	Dimethoate 30 % EC 1.7ml/l-1% Groundnut oil 10 ml/l	Neam Leaf Extract 1500 ppm 2 ml/l

*DAT-Days After Transplanting, ** Zn-3.0%, Fe-2.0%, Mn-1.0%, B-0.50%, Mg-2.0%, →Followed by
